# Emergence of Lyme Disease on the French Riviera, a Retrospective Survey

**DOI:** 10.3389/fmed.2022.737854

**Published:** 2022-03-22

**Authors:** Jacques Sevestre, Antoine Benichou, Vanessa Rio, Pascal Delaunay, Géraldine Gonfrier, Cécile Martaresche, Virginie Carlo, Sarah Nakam, Véronique Mondain, Michel Carles, Pierre Yves Jeandel, Jacques Durant

**Affiliations:** ^1^Laboratory of Parasitology, Nice University Hospital, Nice, France; ^2^IHU Méditerranée Infection, Marseille, France; ^3^Department of Internal Medicine, Nice University Hospital, Nice, France; ^4^Department of Infectious Diseases, Nice University Hospital, Nice, France; ^5^Laboratory of Virology, Nice University Hospital, Nice, France; ^6^SYNLAB Barla GmbH, Nice, France

**Keywords:** Lyme, *Borrelia*, French Riviera, borreliosis, *Ixodes ricinus*

## Abstract

**Background:**

The French Riviera has been declared free of Lyme Borreliosis (LB) for years. Many patients are referred for presumed LB, sometimes with atypical clinical signs and/or doubtful serology, calling the diagnosis into question.

**Methods:**

Patients were assessed for LB diagnosis, depending on clinical presentation, laboratory findings, and further examination by other medical professionals.

**Results:**

Among 255 patients, 45 (18%) were classified as confirmed LB cases [including 28 ongoing LB (10%) and 17 past LB (8%)], and for 210 (82%) a Lyme borreliosis diagnosis was ruled out. Among ongoing LB, 56% had been exposed to or bitten by ticks, exclusively in rural locations of the Alpes-Maritimes. As a result of the diagnostic procedure, 132 (52%) patients had been treated. An alternative diagnosis was established for 134 (52%) patients, covering a wide range of conditions, including mainly psychological (28%) and neurological conditions (25%) or inflammatory and systemic diseases (22%).

**Conclusions:**

Our results strongly suggest the endemicity of LB in the Alpes-Maritimes region. Confirmed LB accounted for 18% of patients while 52% were diagnosed with other conditions.

## Introduction

Lyme disease, also known as Lyme Borreliosis (LB), is the most commonly reported vector-borne disease in the Northern hemisphere, and is caused by spirochaetes of the *Borrelia burgdorferi* genospecies complex (1–3).

Clinicians in Southern France (Alpes Maritimes), relying on historical data, usually consider the condition to be absent in this region for several reasons: reported absence of the vector (*Ixodes ricinus* ticks), due to unfavorable climate, and low number of suspected indigenous cases reported (4, 5).

Clinical manifestations of Lyme borreliosis are wide-ranging, but the most frequent symptom, which is also diagnostic, is a skin rash known as *erythema migrans* (EM). The non-specific nature of many LB clinical manifestations poses a diagnostic challenge, especially in atypical or late forms of the disease. In France and elsewhere, the definition of Lyme disease is a controversial issue, as non-characteristic presentations with symptoms such as fatigue, memory or concentration impairment, headache, arthralgia, and myalgia attributed to LB are on the rise. In this context, LB is often suspected in patients with atypical clinical presentation. Such patients should be referred to reference centers, prior to any presumptive treatment, to investigate for alternative diagnoses (6, 7), and thus avoid over-treating.

## Patients and Methods

We recorded all patients referred for presumed LB to the Lyme disease competence center within the infectious diseases department of Nice University Hospital, France, between January 2016 and January 2020. Patients above 16 years of age with a diagnosis of LB, as presumed by the patient, the general practitioner or a specialist were included. We excluded patients lost during standard clinical follow-up. Variables of interest included age, gender, tick exposure, tick bite, history of EM, LB serology assay results, and past LB treatments. Prior presumed EM was only retained in case of an accurate description. Reasons for referral were classified according to the medical referral letter and patient's complaint as: EM, tick bite, positive or uncertain serology, neurological, rheumatologic (notably arthritis) or polymorphic symptoms, or other. EM was defined as a circular, non-pruriginous erythematous macule with centrifugal extension. Neurological symptoms comprised meningoradiculitis and headaches. Moreover, we defined arthritis as mono- or oligoarthritis, without fever. Personal medical history was reviewed. Signs and symptoms were recorded after thorough physical examination. Symptoms were grouped according to the main organ system involved or classified as polymorphic post-tick bite symptoms (PTBS) when patients had multiple-symptom complaints (at least three symptomatic organs involved and chronic fatigue). Results of LB serology assays, i.e., enzyme-linked immunosorbent assay (*ELISA, Vidas Lyme Biomerieux IgG, and IgM*), and western-blot (*WB: Immunoblot-Mikrogen IgG and IgM*), were collected. They were considered doubtful if not strictly negative. Two-tier serological testing was considered positive when both ELISA and western-blot testing were IgG positive, accordingly to the French guidelines (4). When patients faced a high degree of suspicion of LB, treatment was prescribed according to guidelines depending on clinical presentation. When LB suspicion was intermediate or low, patients were referred to other specialists (neurologist, rheumatologist, internal medicine specialists, psychiatrist or psychologist) in a coordinated pathway to complete investigations. If no differential diagnosis was made, LB presumptive treatment was offered regardless of potential prior treatment, in accordance with the French High Council of Public Health (Haut Conseil de la Santé Publique) guidelines (7). Unexplained, prolonged symptoms such as fatigue, impaired memory or concentration, headaches, arthralgia, and myalgia were not considered characteristic of LB but presumptive treatment was offered or if no alternative diagnosis was reached. Patients were treated with either amoxicillin (50 mg/kg/d) in case of EM, ceftriaxone (2 g/day) in case of neurological involvement, or doxycycline (200 mg/day) in other instances and in case of beta-lactam intolerance for at least 28 days (except in case of EM where the duration was 15 days). Recovery was defined as a return to the patient's previous health state before symptom appearance. No consent form was collected in this context of non-prospective, standardized patient management performed accordingly to national guidelines. Authorization for data processing was obtained. Cases were classified as either ongoing LB, past LB or non-LB. Categorical variables were compared using chi-square with Fisher's exact test. Calculation of Odds Ratio with interval confidence were calculated for all nominal categorical variables. Continuous variables were compared using the Mann-Whitney test. Differences were considered statistically significant for a *p*-value < 5%. Statistical analyses were performed using Statview version 5.0 (SAS institute, USA, Cary, NC, USA).

## Results

Between January 2016 and January 2020, 305 patients presented at the outpatient department for presumed LB. Fifty patients were lost during follow-up. Among the 255 patients analyzed, median age was 50 years (range 18–88) with 62% women (*n* = 158). Patients' characteristics are detailed in Table 1. Forty percent of patients (*n* = 102) had already been treated for LB. The most frequently prescribed antibiotics were Doxycycline (*n* = 50, 49%), Amoxicillin (*n* = 36, 35%), Ceftriaxone (*n* = 5, 5%) and other antibiotics (*n* = 4, 4%). Seven patients had received ineffective treatments (anti-viral, anti-fungal or herbal agents). Fourteen patients had received a second-line LB treatment before the first visit in our referral clinic (6%).The ELISA test was positive in 121 patients (47%) and doubtful in 24 patients (9%). Twenty-six patients had positive ELISA associated with doubtful WB (10%). The two-tier serology (ELISA and WB) was positive in 56 patients (22%). A total of 132 patients (52%) were presumptively treated.

**Table 1:** Patients characteristics.

**Variable**	**Patients (%)**	
Female	158	62%
Age, median (years)	50.00 (18–88)
Tick exposure	220	86%
History of tick bite	159	62%
**History of EM**
Yes	42	16%
Doubtful	26	10%
**Place of tick exposure or bite**
Alpes-Maritimes	107	42%
Other regions	71	42%
Foreign country	28	11% 2%
Var	11	4%
Corse	3	1%
**Reason for referral**
Polymorphic symptoms	104	41%
Tick bite	56	22%
Neurological symptoms	45	18%
Rheumatological symptoms	20	8%
Doubtful serology	16	6%
Other	14	5%
**Clinical signs**
PTBS	104	41%
Neurological	52	20%
Rheumatological	36	14%
Asymptomatic	33	13%
EM	12	5%
Others	18	7%
Median symptom duration, months	Range 15 (0–480)
Anterior LB treatment	102	40%
More than one prior LB treatment	14	5%
Positive two-tier serology	56	22%
Treated patients	132	52%

According to the applied criteria (Table 2), 45 patients were classified as confirmed LB cases (18%), including 28 (11%) with ongoing LB who were successfully treated (12 with EM, 10 with Neuro-Borreliosis and 6 with arthritis) and 17 (7%) with past LB (16 with EM and 1 with Neuro-Borreliosis) who had recovered after prior treatment.

**Table 2:** Lyme Borreliosis diagnostic criteria.

Confirmed Lyme	Ongoing Lyme^*^	4/4 criteria: - Tick bite or exposure - Positive two-tier serology - Characteristic clinical signs - Good response after presumptive treatment
	Past Lyme	4/4 criteria : - Tick bite or exposure - Confirmed prior EM or characteristic clinical signs - Recovery after prior treatment - Absence of symptoms

* *Clinical EM was considered as “Ongoing LB” without additional criteria*.

Patients' diagnostic details are shown in Table 3 and final diagnoses detailed in Table 4. Comparison of Lyme and Non-Lyme groups appears in Table 5. Twenty-five patients supposedly contracted Lyme disease in the local rural area (indigenous cases). In 26 patients, no differential diagnosis could be ascertained despite investigations. Six patients were referred for doubtful serology with no treatment history nor EM and were asymptomatic, none were treated, they were considered as exhibiting a potential post-exposure serological scar or a false-positive serology. Among the 132 (52%) presumptively treated patients, following or concurrently with investigations, 72 achieved a significant response or recovery (54.5% of treated patients), 28 with ongoing LB and 44 with no proven LB.

**Table 3:** Diagnoses among 255 subjects.

**Diagnostic category**			**Number (%)**
**Confirmed Lyme** *n* = 45, (18%)	**Ongoing Lyme** *n* = 28, (11%)	EM	*n* = 12, (5%)
		Neurological	*n* = 10, (4%)
		Arthritis	*n* = 6, (2%)
	**Past Lyme** *n* = 17, (7%)	EM	*n* = 16, (6%)
		Neurological	*n* = 1, (0.5%)
**Non-Lyme** n = 210, (82%)	Psychological disorders		*n* = 38, (15%)
	PTBS		*n* = 36, (14,%)
	Neurological diseases		*n* = 33, (14%)
	Inflammatory and systemic diseases		*n* = 30, (11%)
	Various illnesses		*n* = 23, (9%)
	Other infection		*n* = 10, (4%)
	Other^*^		*n* = 8, (3,%)
	Serological scar		*n* = 6, (2,5%)
	Unknown diagnosis		*n* = 26, (10%)

*EM, Erythema migrans; PTBS, polymorphic post-tick bite symptoms*.

* *Neurological symptoms in 4 patients and rheumatological symptoms in 4 patients*.

**Table 4:** Alternative diagnosis when achieved.

**Non-Lyme differential diagnosis when reached**, ***n*** **=** **134**			
**Psychiatric disorders** ***n*** **=** **38 (28%)**	**Neurological diseases** ***n*** **=** **33 (25%)**	**Inflammatory and systemic diseases** ***n*** **=** **30 (22%)**	**Various illnesses** ***n*** **=** **23 (18%)**	**Other infections** ***n*** **=** **10 (7%)**
Anxiety disorder *n* = 4 Depression *n* = 15 Psychosis *n* = 2 Somatoform disorder *n* = 12 Other *n* = 5	Periph. neuropathy *n* = 5 Multiple sclerosis *n* = 4 Vascular leucopathy *n* = 3 ALS *n* = 2 Parkinson's disease *n* = 3 Small fiber neuropathy *n* = 3 Narrow lumbar canal *n* = 6 Other *n* = 7	Spondyloarthritis *n* = 8 IBD *n* = 3 Microcrystalline arthr. *n* = 4 Psoriatic arthritis *n* = 2 Rheumatoid arthritis *n* = 4 Undefined arthritis *n* = 4 Sjögren's syndrome=2 Other *n* = 3	Fibromyalgia *n* = 6 Arthrosis *n* = 4 Dermatosis *n* = 6 Cancer *n* = 2 SAS *n* = 2 Other *n* = 3	CMV *n* = 2 EBV *n* = 2 Other *n* = 3 Tuberculosis *n* = 1 TBE *n* = 1 Tibola *n* = 1

*Periph., peripheral; ALS, amyotrophic lateral sclerosis; IBD, inflammatory bowel disease; arthr., arthritis; SAS, sleep apnea syndrome; CMV, cytomegalovirus; EBV, Epstein-Barr virus; TBE, tick-born encephalitis; Tibola, Tick-borne lymphadenitis*.

**Table 5:** Comparison between confirmed Lyme group and Non-Lyme group.

	**Non-Lyme (*n* = 210)**	**Confirmed Lyme (*n* = 45)**	***p***	**Odds ratio (confidence interval)**
Age, mean (±SD), years	49.6 (±16.4)	52.8 (±18.2)	0.24	
Sex, *n*	Female	132 (62%)	25 (55%)	0.45	0.74 (0.4–1.4)
Tick exposure	174 (83%)	43 (96%)	**0.05**	**8.2 (1.9–34.6)**
Tick bite	126 (60%)	35 (78%)	**0.03**	**2.3 (1.1–4.9)**
History of EM	Yes + doubtful	59 (28%)	17 (38%)	0.43	1.6 (0.8–3.0)
Clinical signs	PSTB	86 (41%)	11 (24%)	**0.005**	
	Neurological	44 (21%)	12 (27%)		
	Rheumatologic	25 (16%)	6 (11%)		
	Asymptomatic	33 (12%)	5 (11%)		
	Other	16 (7%)	1 (2%)		
	EM	0 (0%)	10 (22%)		
Duration of symptoms^*^	37.8 (±52.0)	23.9 (±41.1)	0.105	
Prior treatment received	85 (40%)	17 (34%)	0.82	**0.9 (0.5–1.7)**
Positive serology (ELISA + WB)	25 (12%)	31 (69%)	**<0.001**	**13.3 (7.7–34.9)**

*EM, erythema migrans; PSTB, polymorphic symptoms after tick bite*.

* *Duration of symptoms: months, mean (± SD)*.

*Statistically significant p-values appear in bold*.

## Discussion

These data confirm the current and potentially recent endemicity of Lyme disease in the Eastern Alpes-Maritimes, and to a lesser extent, on the French Riviera. Recently, Sevestre et al. revealed the current endemicity of *Ixodes ricinus* in various locations and the existence of biotopes compatible with their development and proliferation. Moreover, a significant proportion of the *I. ricinus* ticks collected were found to harbor Lyme group *Borrelia*. Excluding past confirmed cases of LB that were mostly referred for positive or uncertain serology and remained asymptomatic after EM (*n* = 17, 7%), ongoing confirmed LB accounted for 11% of our cohort. These results are consistent with French literature data where a final diagnosis of ongoing LB was reported in 10% of suspected cases in Paris (6), 12% in Besançon (8) and 15% in Nancy (9). A comparable rate in a low prevalence area such as the Alpes-Maritimes may be due to various factors: underestimated endemicity of the disease as mentioned above, population movements (44% of patients had been exposed or bitten by ticks in other regions or in foreign countries) and a recruitment bias. We hypothesize that in an area considered free of LB, local practitioners are less likely to suspect Lyme disease, resulting in a higher ratio of positive to suspect patients. Among our cohort, 210 patients were considered non-LB cases (82%). A differential diagnosis was made in most patients (*n* = 134, 81% of non-LB, i.e., 52.5% of the cohort), with a wide range of conditions, comparable to those presented by Jacquet et al., thanks to a dedicated multidisciplinary care pathway (9). Non-LB final diagnoses mainly consisted in psychological disorders (28%), neurological conditions (25%) or inflammatory and systemic diseases (22%). Some non-LB patients were presumptively treated while undergoing diagnostic investigations (*n* = 60, 36%) as empirical treatment of suspected LB could be considered innocuous and an alternative diagnosis was not obvious. Fourteen patients (23%) presented with moderate, unsustained improvement, potentially induced by a placebo effect (10). The remaining patients showed no improvement (*n* = 46, 77%). No final diagnosis could be reached in 26 patients (10%), consistently with previously cited reports (6, 8, 9).

Definition of possible LB cases remains a major subject of debate. Reports from seronegative, chronically ill patients, with proven Borreliosis and non-typical symptoms who recovered after empirical treatment challenge the conventional discourse around the disease (11). The diagnostic workup for LB traditionally involves assessing the patient's risk, objective signs and symptoms (such as EM), supported by laboratory findings when appropriate (12, 13). Formal serology results in patients presenting with potential LB symptoms may not be sufficient to definitely rule out the disease. Positive serology can be delayed in early Lyme, or remain negative in late forms of the disease, presumably due to suboptimal test sensitivity, even though this appears to be rare (14, 15). Extrinsic factors, including early antibiotic exposure, have likewise been found to influence the humoral response (16), as well as intrinsic factors such as immunodepression or inefficient host immune response (17). False negative serology have been proposed to be due to sequestration of *Borrelia* antibodies in immune complexes (18). Other biological diagnostic methods are under investigation, including culture and molecular biology methods (19–21). Analysis of host antigen-specific T-cell response to *Borrelia* is not widely adopted in clinical practice (22–24).

It is to be noted that ruling out psychological disorders in the context of such clinical presentations can be challenging as patients with “chronic Lyme disease” have been proposed to share identical characteristics of somatoform signs with fatigue, chronic pain and neurocognitive disorders such as memory or concentration impairment (25). Apart from EM, patients with disseminated infection experience signs and symptoms that depend on multiple factors such as geography (26, 27), host predisposition (28), treatment history (16–29) and infecting species. The number of genospecies belonging to the Lyme borreliosis complex continues to expand, while the pathogenicity and the variety in clinical presentations induced by many of them remains unknown (30).

This study has several limitations due to its monocentric design and to several practical difficulties. Moreover, for some patients data concerning prior treatments were obtained from patient interview, which includes a recall bias to our study. Essential work-up assessments such as psychiatric evaluation could not be performed in all patients, partly due to a lack of resources, leading to difficulties in interpreting results. Similarly, delayed investigations may have led us to over-treat certain patients. It is also to be noted that no conclusions may be drawn from empirical antibiotic treatment of suspected Lyme patients, as numerous research works have demonstrated that many improve on placebo treatments.

However, to the best of our knowledge, this work is the first to highlight the current endemicity of clinical Lyme disease. Moreover, our findings are corroborated by a recent study demonstrating the presence of both tick vectors and Lyme group *Borrelia* in tick vectors originating from the Alpes-Maritimes region (31). An alternative diagnosis was reached in over half of referred patients, highlighting the need for multidisciplinary management of these patients. Such a high proportion of patients diagnosed with other conditions could partly be explained by the media coverage of Lyme disease, and controversy or misleading information, resulting in patients or non-expert practitioners overly suspecting LB.

In the end, the present work establishes the occurrence of Lyme borreliosis in the Alpes-Maritimes region, as conforted by recent acarological data (31). We advocate for collaboration between infectious diseases clinicians, microbiologists and acarologists for epidemiological surveillance, and also for multidisciplinary management of patients suspect of Lyme borreliosis, to avoid misdiagnoses and improve patient care regarding this disease.

## Data Availability Statement

The original contributions presented in the study are included in the article/supplementary material, further inquiries can be directed to the corresponding author/s.

## Ethics Statement

Ethical review and approval was not required for the study on human participants in accordance with the local legislation and institutional requirements. Written informed consent for participation was not required for this study in accordance with the national legislation and the institutional requirements.

## Author Contributions

AB, JD, VR, JS, and PJ contributed to the study design and patient clinical evaluation and wrote the manuscript. JS participated to implementation of the research. GG, PD, and CM participated to the analysis of the results. SN and VC performed psychological neurocognitive tests. VM, MC, PJ, and JD supervised the work and reviewed the manuscript. All authors contributed to the article and approved the submitted version.

## Conflict of Interest

CM was employed by company SYNLAB Barla GmbH. The remaining authors declare that the research was conducted in the absence of any commercial or financial relationships that could be construed as a potential conflict of interest.

## Publisher's Note

All claims expressed in this article are solely those of the authors and do not necessarily represent those of their affiliated organizations, or those of the publisher, the editors and the reviewers. Any product that may be evaluated in this article, or claim that may be made by its manufacturer, is not guaranteed or endorsed by the publisher.
